# Induced mismatch repair deficiency: a hypothesis for the transient and reversible sensitization of mismatch repair-proficient tumors to Immune checkpoint blockade

**DOI:** 10.3389/fimmu.2026.1965085

**Published:** 2026-10-08

**Authors:** Paulo P. Assumpção, Alessandro Leal, Williams Fernandes Barra, Mário Hiroyuki Hirata, João Vitor Steimbach, Rommel Mario Rodríguez Burbano, Paulo Kassab, Maria Bencivenga, Martín Hernán Bonamino, P&D consortium

**Affiliations:** 1 Núcleo de Pesquisa Oncológicas (NPO), Universidade Federal do Pará, Belém, Brazil; 2 Mass General Brigham Cancer Institute, Harvard Medical School, Boston, MA, United States; 3 Faculdade de Ciências Farmacêuticas, Universidade de Sao Paulo, São Paulo, Brazil; 4 Programa de Terapia Celular e Gênica, Instituto Nacional de Câncer (INCA), Rio de Janeiro, Brazil; 5 Irmandade da Santa Casa de Misericordia de Sao Paulo, São Paulo, Brazil; 6 Dept of Surgery, Dentistry, Pediatrics and Gynecology, Verona University, Verona, Italy; 7 Vice-presidência de Pesquisa e Coleções Biológicas (VPPCB), Fundação Oswaldo Cruz (FIOCRUZ), Rio de Janeiro, Brazil

**Keywords:** cancer immunotherapy, immune checkpoint blockade, microsatellite instability, mismatch repair, mRNA therapeutics, neoantigens, translational immunology, tumor immunogenicity

## Abstract

Immune checkpoint blockade (ICB) has transformed the treatment landscape of multiple malignancies, producing durable responses and long-term survival in subsets of patients with advanced cancer. However, the clinical benefit of ICB remains largely restricted to tumors displaying pre-existing immune activation, high tumor mutational burden (TMB), or deficient mismatch repair/high microsatellite instability (dMMR/MSI-H) status. In contrast, the vast majority of solid tumors retain proficient mismatch repair (pMMR) function, exhibit limited neoantigen diversity, and frequently display an immunologically “cold” tumor microenvironment, resulting in primary resistance to checkpoint inhibition. Here, we propose the concept of induced mismatch repair deficiency (idMMR), a therapeutic strategy aimed at transiently and reversibly suppressing the core mismatch repair proteins MLH1 and MSH2 in pMMR tumors. We hypothesize that temporary impairment of mismatch repair activity may create a controlled hypermutagenic window during which replication-associated insertion and deletion events accumulate within coding microsatellite regions, with the potential to generate frameshift-derived neoantigens that may enhance tumor immunogenicity. Subsequent restoration of endogenous mismatch repair function would restore basal mutagenesis while preserving newly generated antigenic targets for immune recognition. Unlike constitutive mismatch repair deficiency, idMMR would be pharmacologically controlled and potentially reversible through RNA-based therapeutic approaches. We discuss the biological rationale supporting this hypothesis, the potential immunological consequences of transient mismatch repair suppression, major safety considerations, mechanisms of resistance, and key experimental studies required for validation. Although several critical assumptions remain untested, idMMR may represent a novel framework for converting immunologically cold tumors into ICB-responsive tumors.

## Introduction

Immune checkpoint blockade (ICB) has fundamentally altered the therapeutic landscape of cancer. Antibodies targeting programmed cell death protein 1 (PD-1) (1, 2), programmed death ligand 1 (PD-L1) (3, 4), and cytotoxic T-lymphocyte-associated protein 4 (CTLA-4) (5, 6) have demonstrated durable clinical benefit across a broad range of malignancies. More recently, additional inhibitory pathways, including lymphocyte activation gene-3 (LAG-3) (7, 8), have emerged as clinically relevant targets, further expanding the scope of cancer immunotherapy.

Despite these advances, the clinical success of ICB remains highly heterogeneous. While some patients experience durable responses lasting years, most tumors demonstrate either primary resistance or only transient benefit (9). One of the strongest predictors of responsiveness to checkpoint blockade is the presence of deficient mismatch repair (dMMR) and high microsatellite instability (MSI-H), a molecular phenotype characterized by elevated tumor mutational burden (TMB), abundant neoantigen generation, and pre-existing immune infiltration (10).

The mechanistic basis for this association is increasingly understood. Loss of mismatch repair function permits the accumulation of insertion and deletion mutations within microsatellite regions, many of which occur within coding sequences (11). These alterations generate frameshift-derived neoantigens that are recognized as non-self by the immune system. Consequently, dMMR tumors frequently display increased infiltration by cytotoxic CD8+ T cells, enhanced interferon signaling, activation of antigen presentation pathways, and adaptive upregulation of immune checkpoint molecules, including PD-L1 (12). Together, these features create a tumor microenvironment particularly susceptible to immune checkpoint inhibition.

The clinical implications of this biology have been substantial. Multiple clinical trials have demonstrated remarkable activity of anti-PD-1 therapies in dMMR/MSI-H malignancies, ultimately leading to the first tissue-agnostic regulatory approvals based on a molecular biomarker rather than tumor histology (13).

In contrast, the vast majority of solid tumors remain mismatch repair proficient (pMMR) and microsatellite stable (MSS) (14). These tumors generally exhibit lower mutational burdens, reduced neoantigen diversity, limited T-cell infiltration, and a predominance of immunosuppressive cellular populations within the tumor microenvironment. As a result, response rates to checkpoint blockade remain substantially lower in pMMR tumors, representing one of the major unresolved challenges in contemporary cancer immunotherapy (14).

Current efforts to overcome this limitation have focused on increasing tumor immunogenicity through combinations of chemotherapy, radiotherapy, targeted therapies, epigenetic modulators, oncolytic viruses, cancer vaccines, and adoptive cellular therapies (15). While these approaches have shown varying degrees of success, none have consistently reproduced the exceptional responsiveness observed in naturally occurring dMMR tumors.

This observation raises an important conceptual question: could selected immunological features of the dMMR phenotype be transiently recreated in pMMR tumors?

In this article, we propose the hypothesis of induced mismatch repair deficiency (idMMR), a strategy based on the temporary and reversible suppression of core mismatch repair proteins. Rather than permanently altering the tumor genome, idMMR seeks to generate a controlled and time-limited window of mismatch repair impairment, potentially increasing neoantigen generation and immune recognition while minimizing the long-term consequences associated with constitutive genomic instability. We discuss the biological rationale for this approach, its potential advantages and limitations, and the experimental framework required to evaluate its translational feasibility.

## Why dMMR tumors respond to immune checkpoint blockade

The remarkable sensitivity of deficient mismatch repair (dMMR) tumors to immune checkpoint blockade has established mismatch repair status as one of the most robust predictive biomarkers in modern oncology (14, 16). Although this association was initially attributed to elevated tumor mutational burden (TMB), accumulating evidence indicates that the biological relationship between mismatch repair deficiency and immunotherapy responsiveness is considerably more complex.

The defining molecular feature of dMMR tumors is the inability to correct replication-associated insertion and deletion events that arise within repetitive DNA sequences, particularly microsatellites (14). Although most microsatellites are located in non-coding regions of the genome, a subset is embedded within protein-coding genes. In the absence of functional mismatch repair, replication slippage at these coding microsatellites frequently results in frameshift mutations that alter the downstream reading frame and generate novel peptide sequences absent from the normal human MHC-restricted proteome (17). Unlike many missense mutations, which differ from their wild-type counterparts by only a single amino acid substitution, frameshift mutations can produce extended stretches of entirely non-self amino acid sequences (18). These frameshift-derived peptides are considered particularly attractive targets for immune recognition because they exhibit minimal central immune tolerance and may generate highly immunogenic neoantigens. Consequently, the exceptional sensitivity of many dMMR tumors to immune checkpoint blockade may reflect not only an increased mutational burden, but also the preferential generation of this distinctive class of frameshift-derived neoantigens.

Importantly, the immunological consequences of mismatch repair deficiency extend beyond neoantigen generation alone. Multiple studies have demonstrated that dMMR tumors frequently exhibit dense infiltration by CD8+ cytotoxic T lymphocytes, activation of interferon-responsive transcriptional programs, enhanced antigen presentation machinery, and increased expression of immune checkpoint molecules, including PD-L1 (19). These observations suggest that mismatch repair deficiency contributes not only to antigenicity but also to broader remodeling of the tumor microenvironment.

The relationship between mutation burden and immune responsiveness is not absolute. Many tumors with elevated TMB fail to respond to immunotherapy, whereas some tumors with relatively modest mutation burdens remain sensitive (20). Consequently, it has become increasingly clear that the quality, clonality, presentation, and immunogenicity of neoantigens may be as important as their absolute quantity.

One particularly relevant feature of dMMR tumors is the frequent generation of recurrent frameshift mutations within shared microsatellite-containing genes (21). These mutations may emerge early during tumor evolution and therefore achieve substantial clonality within the tumor population. Clonal neoantigens are believed to represent more effective immune targets than subclonal neoantigens because they are expressed by a larger proportion of malignant cells. This concept has emerged as a central principle in contemporary cancer immunology and may partially explain the exceptional responsiveness of dMMR tumors to checkpoint blockade.

Collectively, these observations suggest that the therapeutic vulnerability of dMMR tumors arises from the convergence of multiple biological processes, including enhanced neoantigen generation, increased antigen presentation, inflammatory signaling, T-cell recruitment, and adaptive checkpoint activation. The possibility of reproducing selected aspects of this phenotype in pMMR tumors forms the conceptual basis of the idMMR hypothesis.

## The idMMR hypothesis

We propose that transient and reversible suppression of the mismatch repair proteins MLH1 and MSH2 may create a temporary state of induced mismatch repair deficiency (idMMR) capable of increasing tumor immunogenicity and sensitizing pMMR tumors to immune checkpoint blockade.

The central premise of this hypothesis is that mismatch repair proficiency is not merely a static biomarker but a biological state that may be transiently modulated. If mismatch repair activity can be temporarily reduced, replication-associated insertion and deletion events may accumulate during a defined treatment window, generating novel frameshift-derived neoantigens that would otherwise never arise in pMMR tumors.

Unlike naturally occurring dMMR tumors, which frequently experience lifelong mismatch repair deficiency, idMMR would be intentionally limited in duration. The objective is not to permanently destabilize the tumor genome but rather to create a controlled period of increased mutagenesis sufficient to enhance immune recognition. Restoration of endogenous mismatch repair function following treatment withdrawal would theoretically restore basal accumulation of mutations while preserving previously generated neoantigens as immunological targets.

A critical distinction between idMMR and constitutive dMMR is therefore reversibility. Whereas naturally occurring dMMR tumors continue to accumulate mutations throughout their evolution, the proposed idMMR state would be pharmacologically constrained and temporally controlled. This distinction may have important implications for both efficacy and safety.

Importantly, the hypothesis does not require the generation of thousands of new mutations. It is conceivable that a relatively limited number of highly immunogenic frameshift events could be sufficient to alter immune recognition, particularly if such mutations arise within coding microsatellites that escape nonsense-mediated decay and generate efficiently presented peptides. Whether this threshold can be reached during a clinically practical treatment window remains unknown and represents one of the central questions that future studies must address.

The idMMR model therefore should be viewed not as a proven therapeutic strategy but as a testable biological framework linking transient genomic instability to enhanced tumor immunogenicity.

## Transient induction of the dMMR phenotype: siRNA-mediated knockdown versus competitive mRNA interference

The transient suppression of the DNA mismatch repair (MMR) pathway represents an alternative oncological strategy designed to convert proficient MMR (pMMR) tumors into a temporary deficient state (dMMR). This conversion accelerates the generation of somatic insertion-deletion mutations and frame-shifted neoantigens, ultimately heightening tumor immunogenicity and sensitizing immunologically cold malignancies to immune checkpoint blockade (22). Among the molecular methods engineered to achieve this phenotypic transition, the use of small interfering RNAs (siRNAs) formulated within lipid nanoparticles (LNPs) to selectively deplete the transcripts of core heterodimeric pillars, specifically MutLα (MLH1) and MutSα (MSH2), represents a primary and highly targeted approach (23). However, since MLH1, MSH2 and many other proteins involved in MMR have considerable half-lives, solely impairing translation through could cause undesired lagging between administration and phenotypic transition.

Alternatively, addressing the lag between administration and transition to a mutagenic phenotype, post-translational competitive inhibition utilizing nucleoside-modified mRNAs has emerged as a highly promising avenue to induce the dMMR state. Rather than preventing protein synthesis, this approach introduces synthetic transcripts that code for dominant-negative protein mutants, such as MLH1^D212N^ and MSH2^K675A^, which feature disrupted ATP binding or hydrolysis domains but retain intact dimerization and DNA-binding capabilities. Consequently, these exogenous proteins actively outcompete and inactivate endogenous wild-type complexes on the chromatin backbone. However, both transient strategies present distinct methodological, kinetic, and pharmacological hurdles that dictate their experimental execution and clinical feasibility.

## Biological rationale and mechanistic framework

The proposed mechanism underlying idMMR can be conceptualized as a sequence of interconnected biological events linking transient mismatch repair suppression to immune-mediated tumor elimination.

The first step involves temporary inhibition of MLH1 and MSH2, two central components of the MutLα and MutSα mismatch repair complexes (24). Simultaneous suppression of both proteins is hypothesized to maximize impairment of mismatch recognition and repair, thereby reducing the likelihood of residual proofreading activity through compensatory pathways.

During this period of reduced mismatch repair fidelity, replication-associated insertion and deletion events may accumulate within microsatellite regions. Tumors characterized by high proliferative activity would be expected to experience the greatest mutational impact, whereas slowly proliferating tumors may generate relatively few new alterations. Consequently, tumor-specific proliferation kinetics are likely to represent a major determinant of idMMR efficacy.

A subset of these mutations may occur within coding microsatellites, producing frameshifted transcripts that encode novel peptide sequences. Following proteasomal processing and presentation through major histocompatibility complex class I molecules, these peptides may become accessible to immune recognition.

Thus, the same immune activation that indicates successful recognition of newly generated tumor antigens may simultaneously trigger compensatory mechanisms that limit T-cell effector function. This therapeutic paradox provides a mechanistic rationale for combining neoantigen-generating strategies with ICB. Rather than simply augmenting an already effective immune response, ICB may be required to prevent adaptive immune resistance from rapidly attenuating the newly established antitumor response (9).

Importantly, this proposed sequence remains hypothetical, and multiple biological bottlenecks may limit efficacy at each stage. Newly generated mutations may fail to produce stable transcripts, particularly when frameshift mutations introduce premature termination codons (PTCs) that can trigger nonsense-mediated mRNA decay (NMD). This post-transcriptional surveillance pathway represents a potentially critical barrier to the proposed mechanism, as transcripts carrying PTCs may be selectively degraded before sufficient mutant protein is produced for processing and antigen presentation. Naturally occurring dMMR tumors may partially overcome this constraint through prolonged clonal selection, allowing mutations that generate immunogenic and sufficiently expressed neoantigens to become enriched over time; an acute, transient idMMR state lacks this evolutionary opportunity. Even when mutant transcripts escape NMD, the resulting peptides may not be generated efficiently, may fail to bind patient-specific HLA molecules, or may not be effectively presented to T cells. Furthermore, neoantigens that are successfully presented may remain subclonal and therefore fail to induce uniform tumor clearance. Thus, NMD should be considered a core post-transcriptional bottleneck within the proposed idMMR-to-immunogenicity cascade rather than a secondary consideration.

From a translational perspective, these observations raise the possibility that transient modulation of NMD could enhance the persistence and translation of newly generated frameshift-containing transcripts, although such an approach would require careful evaluation given the broad physiological functions of the NMD pathway. Future studies should therefore determine the extent to which NMD constrains neoantigen generation following transient idMMR and whether selective, temporally controlled NMD modulation can increase the production of immunogenic mutant peptides without compromising cellular fitness or introducing unacceptable off-target effects.

For this reason, the central prediction of the idMMR model is not simply increased mutation burden, but rather the emergence of a transient immunogenic state characterized by measurable changes in neoantigen generation, antigen presentation, T-cell infiltration, and responsiveness to checkpoint blockade. Demonstrating these interconnected biological changes will be essential for validating the translational potential of the approach.

## Major biological challenges and potential failure mechanisms

Despite the biological plausibility of the idMMR concept, several important uncertainties must be considered before its translational potential can be adequately assessed. Indeed, the success of the proposed strategy depends on a sequence of biological events, each of which may represent a potential bottleneck capable of limiting therapeutic efficacy.

The most fundamental uncertainty concerns the kinetics of mutation accumulation following transient mismatch repair suppression. The central premise of idMMR is that a temporary reduction in mismatch repair fidelity may generate sufficient numbers of immunologically relevant mutations to alter tumor recognition by the immune system. However, the minimum duration of mismatch repair deficiency required to achieve this effect remains unknown. It is conceivable that highly proliferative tumors could accumulate substantial numbers of insertion and deletion events during a relatively short treatment window, whereas slowly proliferating malignancies may generate only modest mutational changes. Consequently, tumor-specific proliferation dynamics are likely to represent an important determinant of therapeutic responsiveness, and highly proliferative tumors, as those with high KI-67 indexes, will likely respond better to idMMR.

Equally important is the distinction between mutation generation and neoantigen generation. Most mutations are unlikely to contribute meaningfully to antitumor immunity. For a newly generated mutation to become immunologically relevant, it must occur within a transcribed region, escape RNA quality-control mechanisms, produce a stable peptide, and ultimately be presented through a functional antigen presentation pathway. Furthermore, the resulting peptide must be recognized by a pre-existing T-cell repertoire capable of mounting an effective immune response. Failure at any point along this sequence could substantially diminish the immunological consequences of induced mismatch repair deficiency.

Tumor heterogeneity represents an additional challenge. Naturally occurring dMMR tumors frequently accumulate mutations over prolonged evolutionary timescales, allowing some neoantigens to become highly clonal and thereby enabling more uniform immune recognition. In contrast, mutations generated during a transient idMMR window may initially arise in only a subset of tumor cells and may therefore produce a mosaic, predominantly subclonal neoantigen landscape. This could limit direct immune-mediated elimination of the entire tumor population. However, subclonal neoantigens may also initiate a broader immune response through antigen spreading. T-cell-mediated destruction of tumor cells bearing newly generated idMMR-associated neoantigens could promote local tumor cell lysis and release additional, previously unrecognized tumor antigens, including antigens shared across the underlying clonal tumor population. Subsequent presentation of these antigens could broaden the immune response beyond the initially targeted subclones, potentially enabling immune-mediated clearance of tumor cells that do not harbor the original idMMR-induced mutations. Thus, the therapeutic relevance of idMMR may depend not only on the quantity of mutations generated but also on their spatial distribution, clonality, and capacity to initiate antigen spreading. Determining whether such a clonal cascade occurs following transient idMMR will be an important objective for future studies.

Even in the presence of immunogenic neoantigens, successful immune recognition cannot be assumed. Numerous tumors evade immune surveillance through disruption of antigen presentation pathways (25). Loss of HLA class I expression, beta-2 microglobulin deficiency, impaired antigen processing, or alterations affecting peptide transport may all limit the capacity of tumor cells to present newly generated neoantigens. Such mechanisms have been extensively described in naturally occurring dMMR malignancies and may similarly restrict the effectiveness of induced mismatch repair deficiency (10, 14).

Finally, it is important to recognize that successful immune activation may itself create new evolutionary pressures. Immunoediting represents a well-established consequence of effective antitumor immunity, whereby immune-mediated selection favors the emergence of resistant tumor clones. Similarly, prolonged antigen exposure may contribute to progressive T-cell dysfunction and exhaustion. Although idMMR is designed as a transient intervention, the extent to which newly generated antigenic landscapes might influence long-term immune dynamics remains uncertain. These considerations highlight the need to view idMMR not as a guaranteed mechanism for overcoming immune resistance, but rather as a strategy that may transiently increase the probability of productive immune recognition.

## Safety considerations and risk mitigation

Any therapeutic strategy based on intentional disruption of genomic maintenance mechanisms must be accompanied by careful consideration of potential safety risks. Unlike conventional immunotherapies, which primarily modulate immune function, idMMR seeks to transiently alter the fidelity of DNA replication itself. Consequently, the possibility of unintended genomic consequences represents one of the most important challenges facing the development of this approach.

The strongest biological precedent for evaluating these risks derives from naturally occurring mismatch repair deficiency syndromes. Individuals with Lynch syndrome and related hereditary disorders provide compelling evidence that sustained mismatch repair deficiency increases cancer susceptibility through the gradual accumulation of somatic mutations (13, 26). However, these conditions also illustrate an equally important principle: malignant transformation generally requires prolonged exposure to genomic instability and the sequential acquisition of multiple oncogenic events (27). Although such observations cannot be interpreted as direct evidence supporting the safety of idMMR, they suggest that the biological consequences of transient mismatch repair suppression may differ substantially from those associated with lifelong deficiency.

Nevertheless, several theoretical risks deserve careful consideration. The most obvious concern is the possibility that transient hypermutation could generate mutations that confer selective advantages upon tumor cells. While the intended goal of idMMR is the creation of immunogenic neoantigens, the same mutational processes could, in principle, produce alterations associated with enhanced proliferation, metastatic potential, or therapeutic resistance. Although the probability of generating such events during a limited treatment window remains uncertain, this possibility cannot be ignored.

A second concern relates to off-target activity in normal tissues. Because mismatch repair serves as a universal genomic maintenance mechanism, inadvertent suppression within proliferative non-malignant compartments could theoretically induce unwanted genomic instability. Particular attention should therefore be directed toward tissues characterized by continuous cellular turnover, including hematopoietic stem cell populations, intestinal crypt epithelium, and regenerative epithelial compartments. The ability to restrict mismatch repair suppression predominantly to tumor cells will likely represent a critical determinant of clinical feasibility.

These considerations underscore the importance of incorporating risk-mitigation strategies directly into the conceptual framework of idMMR. Tumor-selective delivery systems, transient RNA-based approaches, real-time molecular monitoring, and careful restriction of initial clinical investigations to patients with advanced refractory malignancies may collectively reduce potential risks while preserving therapeutic intent. Ultimately, comprehensive toxicological evaluation will be essential before clinical translation can be contemplated.

## Experimental validation framework

The value of the idMMR hypothesis depends not only on its biological plausibility but also on its capacity to generate experimentally testable predictions. For this reason, a systematic validation strategy should accompany any effort to advance the concept toward translational development.

The first objective should be to establish whether transient suppression of MLH1 and MSH2 can be achieved efficiently and reversibly. These studies must define the magnitude, duration, and kinetics of mismatch repair suppression, as well as the timeline of functional recovery following treatment withdrawal. Demonstrating reproducible restoration of mismatch repair activity will be particularly important, as reversibility constitutes one of the defining features distinguishing idMMR from constitutive mismatch repair deficiency.

Once transient suppression has been established, subsequent investigations should focus on determining whether meaningful genomic consequences arise during the induced mismatch repair-deficient state. High-resolution sequencing approaches could be employed to quantify insertion and deletion frequencies, microsatellite instability, mutational signatures, and overall mutation burden over time. Such experiments would provide the first direct assessment of whether a transient hypermutagenic window can generate measurable genomic alterations within clinically relevant timescales.

The next critical question concerns neoantigen generation. Increased mutation burden alone would not validate the hypothesis unless newly generated mutations give rise to potentially immunogenic peptides. Integration of genomic, transcriptomic, and immunopeptidomic approaches may therefore be necessary to determine whether transient mismatch repair suppression produces antigens capable of being presented and recognized by the immune system.

Ultimately, the central prediction of idMMR is not increased mutagenesis but enhanced immune recognition. Accordingly, preclinical studies should evaluate changes in T-cell infiltration, antigen-specific immune responses, interferon signaling, dendritic-cell activation, and responsiveness to immune checkpoint blockade. These investigations should ideally be conducted in immunocompetent models capable of capturing the dynamic interactions between tumor evolution and host immunity.

Finally, safety assessments must accompany all stages of development. Longitudinal studies examining biodistribution, off-target mutagenesis, genomic stability, and the emergence of resistant clones will be essential for defining the therapeutic window within which idMMR might be safely deployed. Together, these experimental efforts would provide a rigorous framework for determining whether induced mismatch repair deficiency represents a viable strategy for expanding the benefits of cancer immunotherapy beyond the currently limited population of naturally occurring dMMR tumors.

## Discussion

The remarkable clinical success of immune checkpoint blockade has established a new paradigm in cancer treatment (28). However, despite the transformative impact of these therapies, most patients with advanced solid tumors continue to derive limited benefit. This disparity reflects a fundamental biological reality: effective immunotherapy requires the presence of a sufficiently immunogenic tumor microenvironment capable of supporting productive antitumor immune responses. The majority of solid malignancies fail to meet this requirement (29, 30).

Among the various biomarkers associated with responsiveness to checkpoint inhibition, mismatch repair deficiency remains one of the most robust and reproducible predictors of therapeutic benefit (16). Tumors harboring deficient mismatch repair display a unique combination of features that collectively favor immune recognition, including elevated neoantigen burden, enhanced antigen presentation, increased T-cell infiltration, and adaptive expression of immune checkpoint molecules (16). Importantly, these characteristics emerge as a consequence of a specific biological state rather than as independent phenomena.

The central premise of the idMMR hypothesis is that this biological state may be, at least partially, inducible. Rather than viewing mismatch repair status solely as a fixed tumor characteristic, we propose that mismatch repair activity could represent a dynamic and therapeutically modifiable parameter. If transient suppression of mismatch repair function proves capable of generating sufficient immunological consequences, it may become possible to transform tumors that are intrinsically resistant to immunotherapy into tumors that are temporarily susceptible to immune-mediated elimination.

This concept differs fundamentally from many existing approaches designed to increase tumor immunogenicity. Cancer vaccines, adoptive cellular therapies, antibody–drug conjugates (ADCs), and personalized neoantigen-based strategies generally rely on pre-existing tumor antigens or defined molecular targets (31). In contrast, idMMR seeks to generate novel tumor-specific antigens directly within the tumor, thereby modifying its antigenic landscape rather than simply enhancing recognition of existing targets. This feature links idMMR to the concept of *in situ* vaccination, in which the tumor itself becomes the source of antigens for a systemic antitumor immune response.

However, neoantigen generation alone may not be sufficient. Effective *in situ* vaccination requires antigen release in an inflammatory context (32). Therefore, the mode of tumor cell death induced after idMMR may be a critical determinant of therapeutic efficacy. Immunogenic cell death (ICD) involves the release or exposure of damage-associated molecular patterns (DAMPs), including extracellular ATP, HMGB1, and surface calreticulin, which promote dendritic-cell recruitment, maturation, antigen uptake, and cross-presentation (33). If idMMR-induced tumor cells undergo ICD rather than silent, non-inflammatory apoptosis, these signals could facilitate T-cell priming against newly generated neoantigens. This mechanism may complement ADCs, which can induce tumor-cell killing and, depending on their payload and cellular context, contribute to immunogenic tumor-cell death and induce new mutations, although their effects on antigen presentation and systemic immunity require specific evaluation. The targeted delivery of the payload to the tumor could act as a synergistic mechanism with idMMR to further induce neoantigen formation in a site specific fashion, adding another layer of specificity to the approach.

This possibility is particularly relevant because idMMR-generated mutations may be subclonal. Recognition of only these subclonal neoantigens may be insufficient to eliminate the entire tumor. In contrast, ICD-associated cross-presentation could promote antigen spreading, extending the immune response from newly generated neoantigens to additional antigens expressed by neighboring or previously unrecognized tumor clones.

At the same time, it is important to acknowledge that the proposed model remains speculative. Several assumptions underlying the hypothesis have not yet been experimentally validated. The quantity of neoantigens generated during transient mismatch repair suppression, their clonality, their immunogenicity, and their ability to drive meaningful antitumor responses remain unknown. Likewise, the balance between beneficial neoantigen generation and potentially deleterious mutagenesis has yet to be defined. These uncertainties do not invalidate the hypothesis, but they reinforce the need for rigorous experimental investigation before translational applications can be contemplated.

From a broader perspective, the idMMR concept may contribute to an emerging shift in cancer immunology. Increasingly, therapeutic success appears to depend not only on removing inhibitory immune signals but also on actively creating conditions that favor immune recognition (30). The future of cancer immunotherapy may therefore involve a combination of checkpoint blockade with interventions specifically designed to increase tumor visibility to the immune system. Within this framework, transient induction of mismatch repair deficiency represents one possible strategy for overcoming the immunological limitations that characterize many currently refractory malignancies.

## Translational implications for cancer immunotherapy

If validated experimentally, the implications of induced mismatch repair deficiency could extend beyond the immediate context of mismatch repair biology. The concept introduces a broader therapeutic principle in which transient and reversible manipulation of tumor evolutionary processes is used to enhance susceptibility to immune-mediated elimination.

Current immunotherapeutic strategies often focus on strengthening immune effector functions through checkpoint inhibition, cytokine modulation, cellular therapies, or vaccination approaches (28). While these interventions primarily target the immune system itself, idMMR represents an alternative paradigm that targets the tumor as the source of immune invisibility. By temporarily altering the mutational dynamics of tumor cells, the strategy seeks to increase the probability that immunologically relevant targets will emerge and become accessible to immune surveillance.

This perspective may be particularly relevant for tumor types that historically demonstrate limited responsiveness to checkpoint blockade, including microsatellite-stable colorectal cancer, pancreatic adenocarcinoma, prostate cancer, and many forms of breast cancer (9). These malignancies collectively account for a substantial proportion of global cancer mortality and remain among the most challenging diseases in immuno-oncology (34). Even modest improvements in immunotherapeutic sensitivity within these populations could have significant clinical impact.

Importantly, the translational value of idMMR should not be measured solely by its direct clinical potential. Even if the strategy ultimately proves unsuitable for therapeutic implementation, the experimental studies required to evaluate the hypothesis may provide important insights into the relationship between mutation generation, neoantigen evolution, immune recognition, and tumor adaptation. Such knowledge could inform the development of future immunotherapeutic approaches beyond the specific context of mismatch repair modulation.

## Conclusion

The ability of deficient mismatch repair tumors to respond to immune checkpoint blockade has transformed our understanding of the relationship between genomic instability and antitumor immunity. Nevertheless, the vast majority of solid tumors remain mismatch repair proficient and continue to derive limited benefit from currently available immunotherapeutic approaches.

In this article, we propose the hypothesis that transient and reversible suppression of key mismatch repair components may create a controlled hypermutagenic state capable of increasing neoantigen generation and enhancing immune recognition in otherwise immunologically resistant tumors. Unlike constitutive mismatch repair deficiency, the proposed induced mismatch repair deficiency (idMMR) state would be pharmacologically controlled and temporally restricted, potentially allowing the immunological benefits of increased antigenicity to be explored while limiting prolonged exposure to genomic instability.

Although the hypothesis is supported by several established principles of tumor immunology and mismatch repair biology, important uncertainties remain (**Figure 1**). The kinetics of mutation accumulation, the generation of clinically relevant neoantigens, the extent of immune activation, and the long-term safety of transient mismatch repair suppression all require careful experimental investigation. Consequently, idMMR should be viewed not as a validated therapeutic strategy but as a testable conceptual framework designed to stimulate further research.

**Figure 1:**
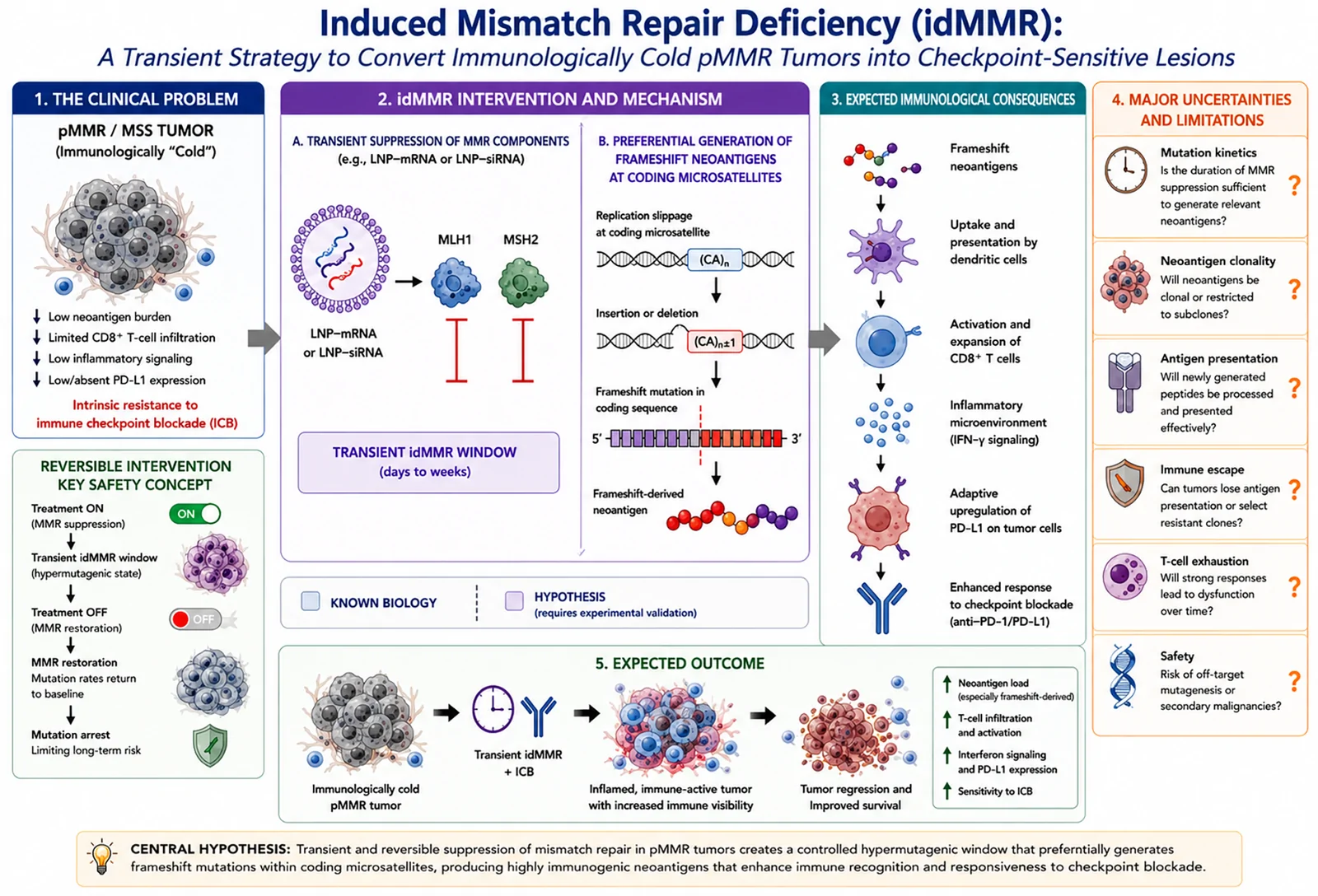
Proposed strategy for induced mismatch repair deficiency (idMMR) to sensitize pMMR/MSS tumors to immune checkpoint blockade. The schematic illustrates the proposed therapeutic rationale for transient and reversible suppression of mismatch repair (MMR) in proficient MMR (pMMR)/microsatellite stable (MSS) tumors. (1) pMMR tumors typically exhibit low neoantigen burden, limited T-cell infiltration, and poor responsiveness to immune checkpoint blockade (ICB). (2) Temporary inhibition of key MMR components (e.g., MLH1 or MSH2) using lipid nanoparticle (LNP)-delivered mRNA or siRNA is hypothesized to create a controlled hypermutagenic window that preferentially generates frameshift mutations within coding microsatellites, leading to the formation of highly immunogenic neoantigens. (3) These neoantigens are expected to promote antigen presentation, CD8^+^ T-cell activation, inflammatory signaling, and increased sensitivity to ICB. (4) Major biological and translational uncertainties, including mutation kinetics, neoantigen clonality, antigen presentation efficiency, immune escape, T-cell exhaustion, and long-term safety, require experimental validation. (5) Following restoration of MMR activity, the proposed outcome is conversion of immunologically cold tumors into inflamed, checkpoint-sensitive lesions while limiting long-term mutational risk. Elements shaded in blue represent established biological knowledge (1), whereas purple-shaded elements denote the proposed hypothesis (2-5).

Ultimately, the significance of the idMMR hypothesis lies in its attempt to address one of the central challenges of modern immuno-oncology: how to render immunologically invisible tumors visible to the immune system. Whether transient induction of mismatch repair deficiency can achieve this objective remains to be determined. However, the hypothesis provides a biologically grounded and experimentally tractable model through which this question can now be systematically explored.

## Data Availability

The original contributions presented in the study are included in the article/supplementary material, further inquiries can be directed to the corresponding author/s.

## Group members of P&D consortium

Geraldo Ishak, João Felipe Acioli, Carolina Baraúna de Assumpção, Paula Baraúna de Assumpção, Naomi Crispim Tropeia, Jessica Manoelli Costa da Silva, Maria Izabel Florindo, José Augusto Pereira Carneiro Muniz, Samir Mansour Casseb, Livia Erika Carlos Marques, Thiago Dominguez Crespo Hirata and André Salim Khayat.
